# Genetic Association Studies Reporting on Variants in the C-Reactive Protein Gene and Coronary Artery Disease: A Meta-Analysis

**DOI:** 10.1097/MD.0000000000001131

**Published:** 2015-08-14

**Authors:** Yujie Shi, Jian Zhang, Chen Tan, Wei Xu, Qi Sun, Junxia Li

**Affiliations:** From the Cardiovascular Diseases Institute, General Hospital of Beijing Military Command of PLA, Beijing, China.

## Abstract

C-reactive protein (CRP) is a commonly used inflammatory marker and elevated CRP levels are shown to increase the risk of coronary artery disease (CAD). Sequence variations in the *CRP* gene believed to influence the protein levels have been extensively investigated in CAD community. Most of the published studies, however, have reported mixed findings. The objective of the present study was to examine the associations of *CRP* variants (+942G>C, −717A>G, +1444C>T) with genetic risk of CAD by use of a meta-analysis.

The human case–control studies were identified through online search, hand search, and contacting the authors of original articles. We performed both random-effect and fixed-effect meta-analysis to estimate CAD risk (odds ratios, OR). This analysis combined 16 studies in total. We found +942G>C was not associated with CAD risk when all data were pooled together, nor did we find a significant association in subgroup analyses. Meta-analysis of +1444C>T studies showed a similar trend. However, a borderline association with CAD risk was revealed for −717A>G (random-effect: OR = 0.53, 95% CI = 0.28–1.00 for the homozygous model; random-effect: OR = 0.51, 95% CI = 0.26–1.00 for the recessive model).

These data suggest that the *CRP* gene variants examined may not modulate CAD risk.

## INTRODUCTION

Coronary artery disease (CAD) is the foremost cause of preventable death across the globe. The number of deaths caused by heart disease has reached to 596,339 in the United States in 2011^1^; such a threatening trend will continue in years to come and CAD-related deaths will account for a large part (∼15%) of total deaths worldwide in 2030.^2^ The concept of inflammation involved in CAD pathogenesis at every stage from onset to development and break of atherosclerotic plaques has been widely accepted. Many groups have set out to characterize the pathological role of biomarkers related to inflammatory reactions in the initiation of CAD,^3–5^ in an attempt to expand the current knowledge on the heterogeneous etiology and molecular mechanism, thereby lowering the incidence of this aggressive disease.

A most commonly used inflammatory marker is C-reactive protein (CRP), an acute phase reactant frequently presenting in atherosclerotic lesions.^6,7^ Alternations in serum levels of CRP have biological significance including reflection of inflammation stages and higher CRP levels predispose individuals to various diseases. A series of clinical studies have shown that upregulated CRP serum levels result in increased likelihood to develop CAD.^8,9^ This observation was recently confirmed in multiple reports, where baseline CRP levels that may be affected by genetic factors were suggested to link with prognosis of cardiovascular diseases and short-term mortality.^10,11^ CRP levels vary substantially between individuals, which suggests an inheritable component in the regulation of serum levels. Available data have documented that an almost half of the variance could be attributable to genetic variants.^12,13^

The human *CRP* gene on chromosome 1q21–23 contains 2 exons. Data on association between the variants at *CRP* locus and the coding of amino acids are currently limited and +942G>C (rs1800947) was previously reported to exert no influence on the amino acid.^14,15^ Several lines of evidence have established a significant connection of genetic variants with CRP levels, including +942G>C, −717A>G (rs2794521), and +1444C>T (rs1130864).^16,17^ The functional properties of these variants prompted a number of investigators to evaluate their contributions to genetic risk of CAD. The published studies, however, have produced mixed findings.^18–20^ More importantly, in a previous genetic analysis published in 2013, Li et al^21^ identified a significant increase in risk of CAD in relation to +942G>C. This effect estimation is possibly incorrect, because 2 papers with more than 2000 subjects were mistakenly categorized into “African” and “Asian,” respectively. Herein, we hospitalized that the aforementioned *CRP* gene variants may be genetic risk factors for CAD. To test the hypothesis, we chose to perform a meta-analysis.

## METHODS

### Search Strategy

A 3-stage literature search was carried out to identify the studies of *CRP* variants and CAD risk. We first searched the Embase, PubMed, Web of Science, CNKI, and CBM databases. We used search terminology (polymorphism) AND (CRP) AND (CAD) and their synonyms (variants, genotypes, ischemic heart disease, *CRP*, SNP, CAD, CHD, and MI) to identify studies. Then we carefully checked the studies analyzed in a meta-analysis for the association of *CRP* variants with CAD risk^21^ and hand searched the citations quoted in the single studies eligible for this meta-analysis. We also contacted the authors of a study with incomplete data in the original article,^22^ although no response was received. The study was approved by the ethics committee of General Hospital of Beijing Military Command of PLA.

### Eligible Studies and Data Abstraction

We selected eligible trials without restraints on a minimum of sample size or language used in publication. Inclusion criteria required the trials:

1. had a case–control design;
2. evaluated the association between genetic risk of CAD and at least one of the *CRP* variants; and
3. published genotype frequency in detail or the data could be obtained after contacting the author.

The studies satisfying all of the predescribed criteria were considered for further analysis. When 2 or more studies contained the same cases, we selected the largest study in which genotype frequency was detailed.

To maximize the accuracy of data, data abstraction was carried out independently by 3 investigators. Characteristics recorded for each trial included genotyped cases and controls, genotype distribution, first author, publication year, study country, racial descent (ethnicity), minor allele frequency in controls, and *P* value for Hardy–Weinberg equilibrium (HWE). Disagreements were resolved via discussion.

### Statistical Analysis

We calculated odds ratios (ORs) along with 95% CIs to evaluate risk of CAD related to *CRP* variants. Calculation of summary ORs was carried out under the homozygous model, dominant model, and recessive model by use of a fixed-effect or random-effect meta-analysis. The extent of heterogeneity was measured by the χ^2^-based Q test. *P* values ≤0.05 were deemed statistically significant.^23^ The I^2^ metric was also used to test the proportion of total dissimilarity across the studies.^24^ I^2^ ≥ 50% indicated large heterogeneity. In a case of significant heterogeneity, the fixed-effect model was applied to summarize ORs for each trial^25^; otherwise, the random-effect model was performed.^26^ Stratified analyses by ethnicity, HWE (studies with or without HWE deviation), and sample size (>200, <200) were performed for *CRP* +942G>C variant. We used forest plots to show the combined results of all studies. HWE was tested for the included studies by a χ^2^ test. Publication bias was determined by 2 analytic tools-Begg's funnel plots and Egger's linear regression analysis.^27^ The leave-one-out sensitivity analysis was performed to check if the single studies had apparent influence on the overall meta-analysis results. The *P* values <0.05 were considered significant unless otherwise stated. All statistical data were analyzed using Stata software (version 12.0, Stata Corp LP, College Station, TX).

## RESULTS

### Meta-Analysis Database

**FIGURE 1:**
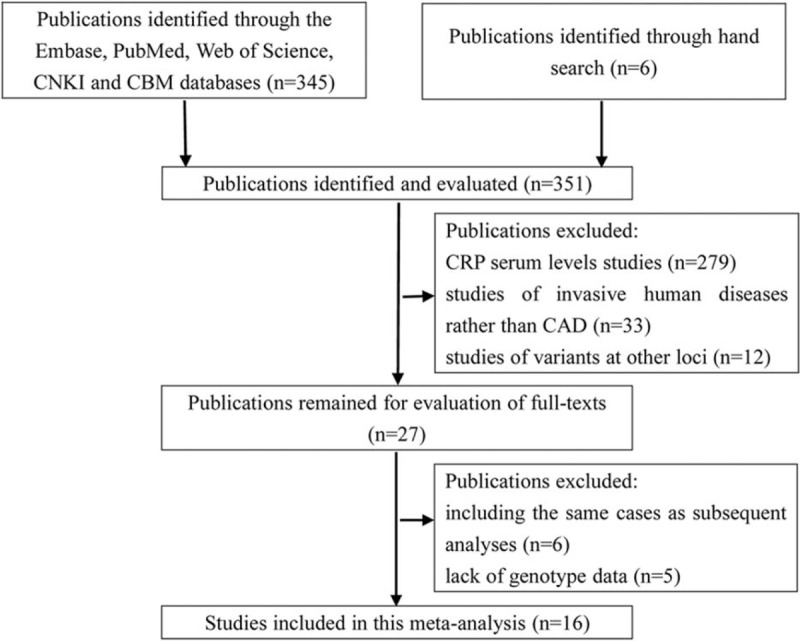
Flow chart for the selection process of the included studies.

Study selection process is graphically represented in Figure 1. We identified a total of 351 reports, of which 335 were discarded due to various reasons (CRP serum levels studies, studies of invasive human diseases rather than CAD; studies of variants at other loci; inclusion of the same cases as subsequent analyses; lack of genotype data). We finally were left with 16 studies.^14,15,18–20,28–38^ As shown in Table 1, 9 studies from the USA, Italy, Croatia, Germany, Iran, Egypt, or India were categorized as Caucasians and the remainders consisting of 7 Asian studies were all conducted in Chinese subjects. There were 14 case–control studies for +942G>C, 5 for −717A>G, and 3 for +1444C>T. The Asian studies had a relatively smaller sample size compared with the Caucasian studies. Deviation from HWE was indicated in the genotype distribution of 4 studies for +942G>C.^20,28,33,35^ Moreover, there was wide difference in genotyping measurement and matching characteristics across studies.

**TABLE 1:**
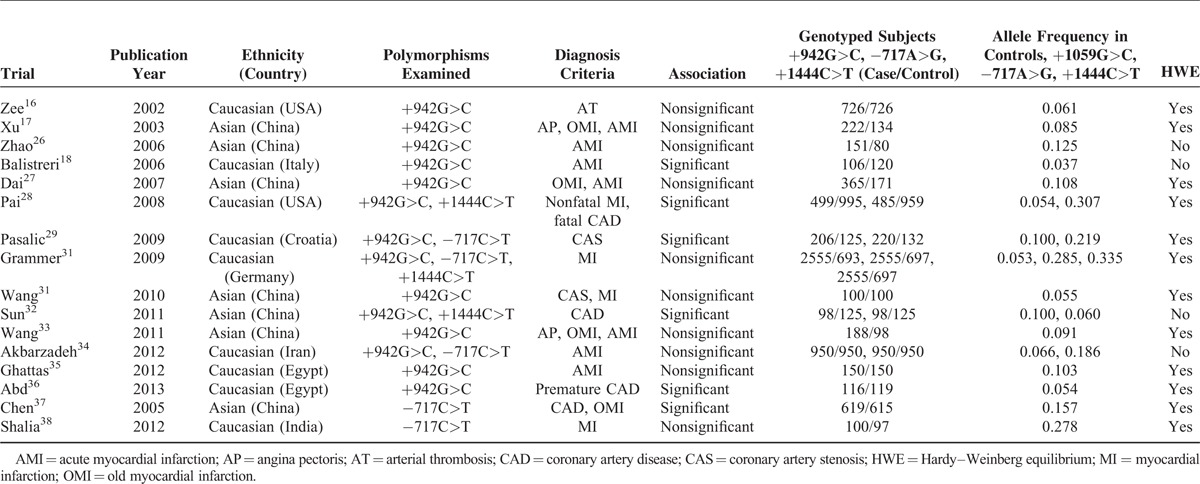
Summary Characteristics of the Eligible Studies

### Quantitative Analysis

**FIGURE 2:**
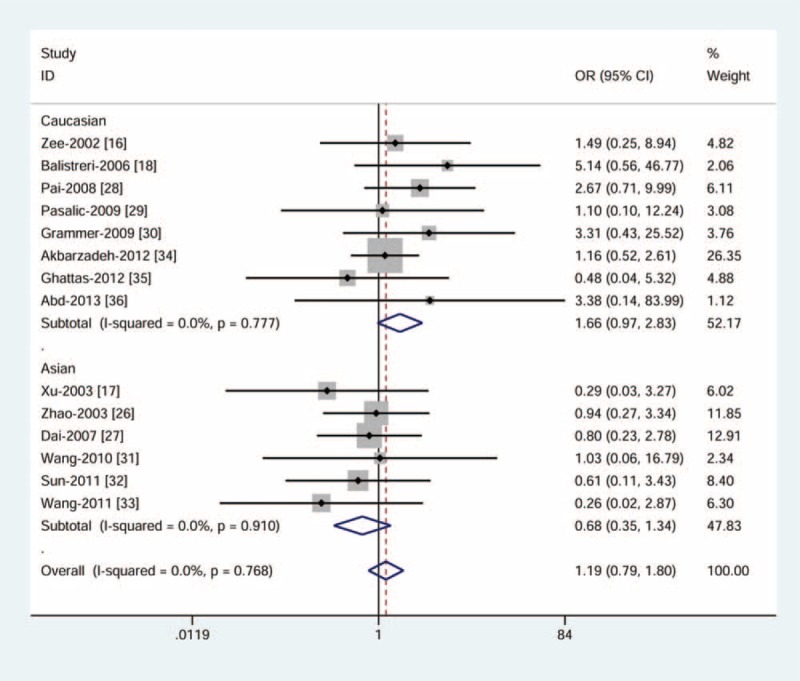
Meta-analysis for the association between *CRP* +942G>C variant and CAD risk by fixed-effect model (homozygous model). The squares and horizontal lines correspond to ORs and 95% CIs of specific study, and the area of squares reflects study weight (inverse of the variance). The diamond represents the pooled ORs and its 95% CIs. CAD = coronary artery disease; CI = confidence intervals; OR = odds ratios.

Table 2 shows the main results of fixed-effect or random-effect meta-analysis. Overall data suggested that individuals harboring the genotypes of +942G>C did not have higher risk of CAD. Subgroup analysis by ethnicity, HWE deviation, and sample size demonstrated a similar nonsignificant trend toward increased risk of CAD. Figure 2 shows the results of all studies under the homozygous model. We observed large between-study heterogeneity in the dominant model (P_h_ = 0.013, I^2^ = 51.6). With the aid of subgroup analysis and sensitivity analysis, we identified the study from the USA was the main cause of nonhomogeneous results.^30^ A significant increase in homogeneity was seen when this study was excluded (P_h_ = 0.115, I^2^ = 33.5). The overall effects were not influenced (data not shown).

**TABLE 2:**
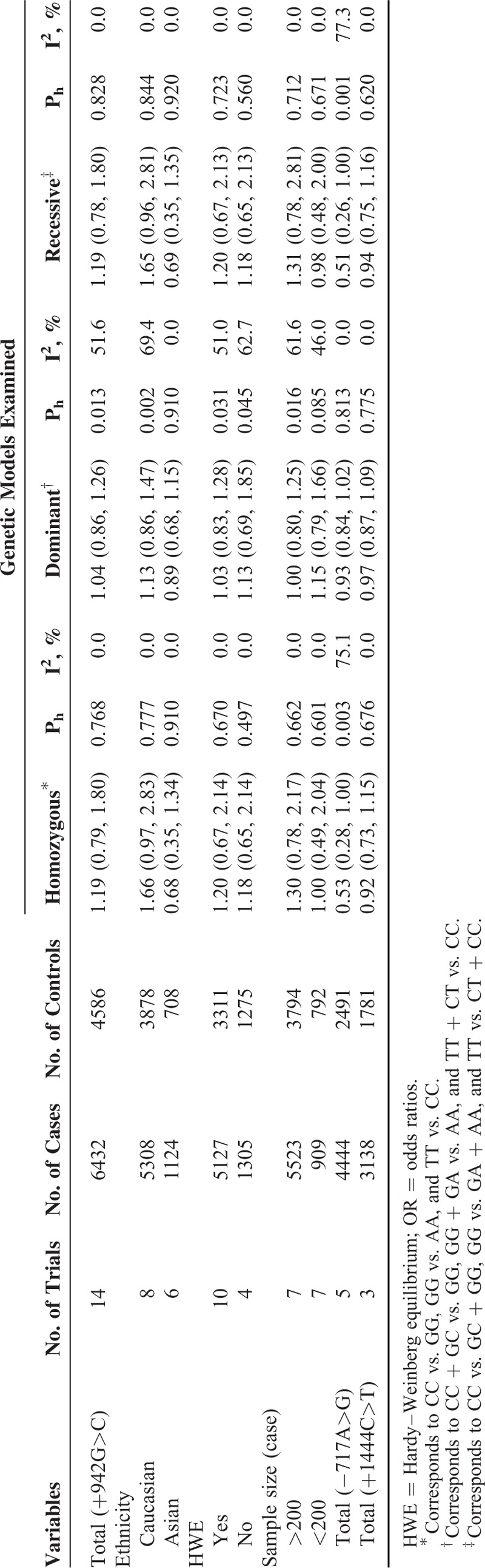
Pooled ORs and Heterogeneity Values (P and I^2^)

**FIGURE 3:**
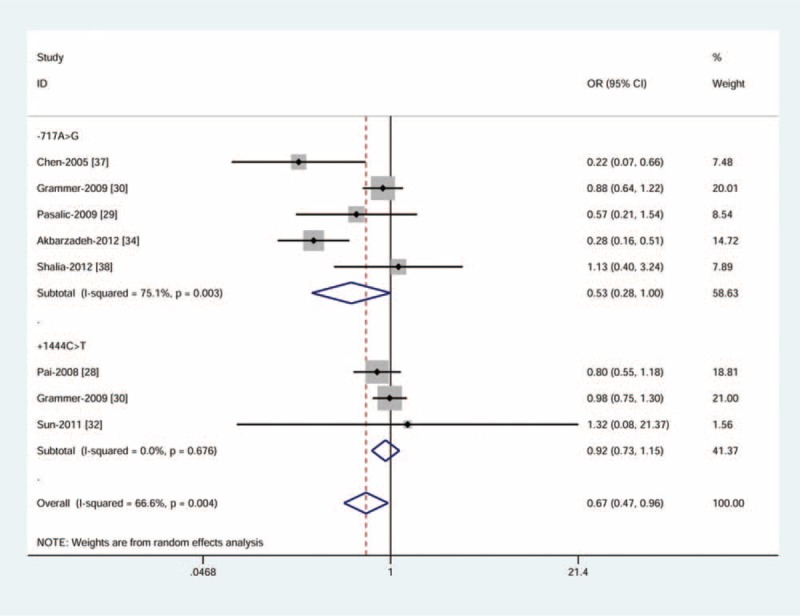
Meta-analysis for the association between *CRP* −717A>G variant and CAD risk by random-effect model (homozygous model). The squares and horizontal lines correspond to ORs and 95% CIs of specific study, and the area of squares reflects study weight (inverse of the variance). The diamond represents the pooled ORs and its 95% CIs. CAD = coronary artery disease; CI = confidence intervals; OR = odds ratios.

For −717A>G variant, we found a borderline reduction in CAD risk under the homozygous model (random-effect: OR = 0.53, 95% CI = 0.28–1.00) (Figure 3) and the recessive model (random-effect: OR = 0.51, 95% CI = 0.26–1.00), indicating the GG genotype was more likely than the AA genotype or the AA and GA genotypes to reduce risk of CAD in general population. We observed notable heterogeneity (P_h_< 0.05, I^2^> 50%) that may attribute to dissimilarities in study design and methodology (Table 2).

Meta-analysis of +1444C>T variant and CAD risk demonstrated no evidence of a significant association. All results were found highly homogeneous (P_h_> 0.05, I^2^ = 0) (Table 2).

### Publication Diagnosis

**FIGURE 4:**
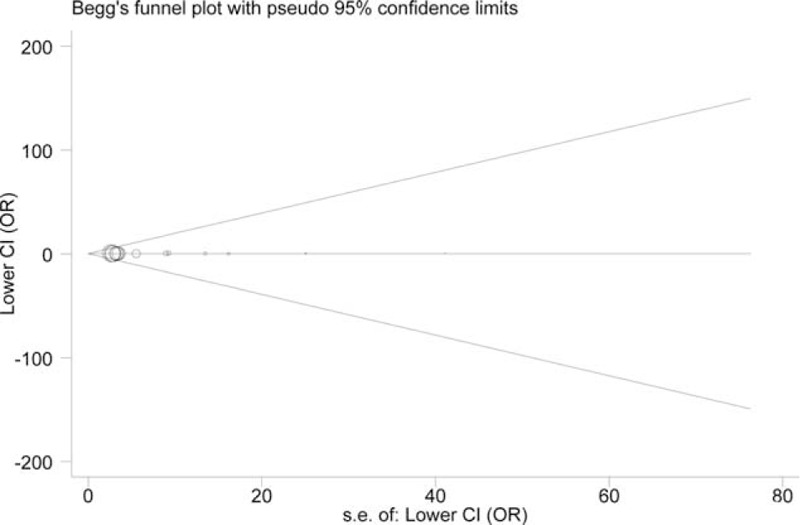
Publication bias test for all included studies for *CRP* +942G>C variant (recessive model). Log OR is plotted versus standard error of Log OR for each included study. Each circle dot represents a separate study. OR = odds ratios.

**FIGURE 5:**
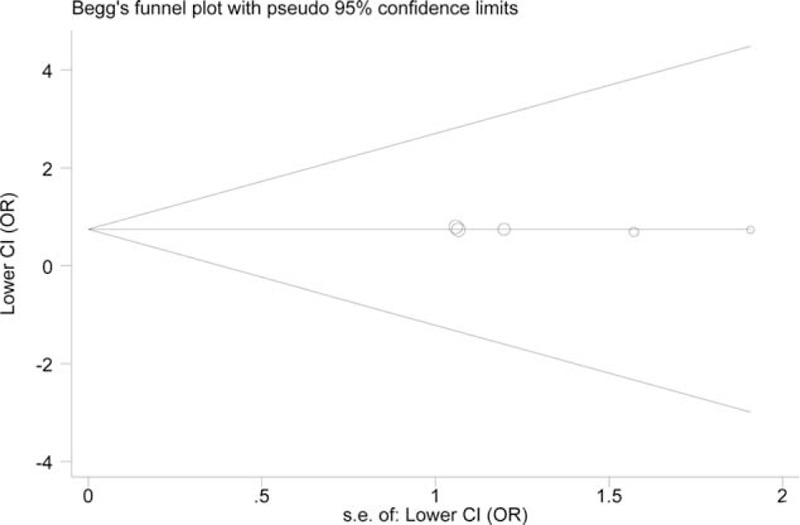
Publication bias test for all included studies for *CRP* −717A>G variant (dominant model). Log OR is plotted versus standard error of Log OR for each included study. Each circle dot represents a separate study. OR = odds ratios.

According to the Begg's test, the funnel plots for +942G>C were symmetric (z = 0.22, *P* = 0.827 for the recessive model) (Figure 4). The symmetry was confirmed by the Egger's test (z = −0.05, *P* = 0.959). Similarly, the tests presented no evidence of notable publication bias in the trials for −717A>G (Begg: z = 0.73, *P* = 0.462; Egger: z = −1.20, *P* = 0.315 for the dominant model) (Figure 5).

## DISCUSSION

Inflammation is believed to associate with elevated cardiovascular susceptibility. The CRP is a well-characterized inflammatory biomarker that has been established as a genetic determinant for a variety of immune-inflammatory diseases. Epidemiological and molecular data on higher CRP levels and increased risk of acute coronary ischemia, angina pectoris, and MI illustrate a clear relationship between *CRP* and cardiovascular events.^39–41^ The genetic variations in the *CRP* gene, such as +942G>C and −717A>G, have been shown to play a big part in the plasma level changes.^35^ The minor allele of +942G>C was found to lower CRP levels,^30^ a finding that seems to support a protective effect of this common variant against the development of CAD as reported in a case–control study in which a significant association of lower plasma CRP level with +942C allele is demonstrated.^14^ Such a protective role +942G>C plays in CAD has been further evidenced in multiple molecular studies from UK, Finland, and the USA.^42–44^ These interesting findings indicate that +942G>C genotypes may protect against the malignant progression of CAD through decreasing the serum levels of the protein.

An involvement of −717A>G in CAD was first identified in a British study.^42^ However, current knowledge of how this promoter region variant affects its gene level remains quite limited, due to the considerable discrepancy in earlier observations. A study from the USA employed 3 apparently healthy populations suggested that −717A>G is unrelated to CRP levels, even though it is strongly associated with a decrease in CAD risk.^45^ Interestingly, according to Pasalic et al, −717A>G has no contributions to CRP levels in CAD patients, but an increase in the plasma level is discovered in healthy Croatian subjects with the −717G allele.^14^ Therefore, the biological role of −717A>G in CRP function and CAD risk remains to be elucidated.

Since sequence variants of the *CRP* gene are important determinants of its serum levels, the effects of these variants on CAD development have received widespread attention in recent years. Zee and Ridker^18^ investigated a single variant (+942G>C) and found no association with risk of CAD, and since then the association of common *CRP* variants with CAD risk has come under scrutiny. Replication efforts for the association with CAD include Xu et al^19^, who provided evidence supporting no contribution of +942G>C to CAD. Although this finding is consistent with the original study, it is challenged by a pilot case–control study, where the authors found +942C is associated with approximately 4-fold higher risk of CAD.^20^ For −717A>G *CRP* gene variant, published studies have shown the same contradictory results. According to Chen et al^37^, carriers of Chinese ancestry carrying −717A are genetically predisposed to CAD, which appears to contradict a study from Germany.^31^ In this work, Grammer et al analyzed all of the variants being investigated in this meta-analysis and detected no relationship between the genetic variants, previously shown to affect circulating CRP, and the prevalence of CAD. Several plausible reasons can explain the substantial inconsistency in these observations, including distinct genetic background, various errors in methodology, and nonstandard selection of control subjects. For example, Grammer et al, unlike the other investigators who employed healthy individuals as controls, considered patients with stenoses <20% as controls; this classification may lead to imprecise assessment of the association.^31^

In view of these mixed findings, we were spurred on to evaluating the relationship between *CRP* variants and CAD risk by means of meta-analysis. A total of 16 articles comprised of 14 studies for +942G>C, 5 for −717A>G and 3 for +1444C>T were summarized in this analysis. For +942G>C *CRP* gene variant, we performed both global analysis and stratified analyses, with none of the analyses indicating a significant genetic association. This lack of an association with CAD persisted when data for −717A>G and +1444C>T were analyzed. These findings fail to confirm the previous epidemiological and meta-analysis data supporting a strong association between the *CRP* variants and incident CAD. Li et al^21^ analyzed 6751 Caucasians and found increased CAD risk associated with +942G>C genotypes. Conversely, we failed to replicate this significant association by use of a meta-analysis combining 9186 Caucasians. The null association indicated in the present study highlights the importance of a sufficient sample size to derive a precise evaluation of a genetic association. Therefore, whether the *CRP* gene variants act as modifiers of CAD risk remains to be validated in a large-scale study.

The role of genetic variants in the carcinogenesis of cardiovascular disease has received widespread attention in recent years. For example, Lanni et al^46^ targeted the Pl^A1/A2^ variant of *GPIIIa* and found that the Pl^A2^ allele is a risk factor of ischemic stroke. In a recent study, presence of the same variant was reported to increase the risk of stroke and MI.^47^ Many other candidate gene polymoprhisms, such as *CaMK4* and *GRKs*, are frequently shown to induce hypertension.^48,49^ These findings suggest that variants are likely to play a pivotal role in the development of cardiovascular disorders. The null results shown in the present work need to be verified in a new study, possibly in a sufficient number of subjects.

In this analysis, multiple factors may limit the extent the results can be generalized to. First, significant heterogeneity was indicated across the studies for +942G>C and −717A>G. We identified the major sources of heterogeneous results, and noted that the combined effects remained stable when the outlawed study was excluded, suggesting the impact of heterogeneity on overall estimates exists but not substantial. Second, many previous studies support an association of *CRP* variants with incident CAD, which nevertheless is not confirmed in the present study. It is possible that *CRP* variants are low-penetrance polymorphisms and the minor effects on common diseases require a sufficiently large study to detect. Third, several studies of +942G>C deviated from HWE, and it is the deviation that may affect the precision of overall meta-analysis results. Fourth, CAD is genetically heterogeneous. In addition to inherited genetic factors, lifestyles and exposure to environmental risk factors are important components in the malignant progression. Further research is clearly required to investigate the pathogenesis of this common cardiovascular disorder. Finally, this article only focused on the gene effect on susceptibility to CAD. The effect of the disease modifying (complications, type of onset, and severity) or responsibility to the medication was not studied.

This analysis also has several strengths. The first strength refers to the novel findings not discovered in earlier quantitative assessment, including the null association of +942G>C and CAD risk in Caucasians, and lack of a relation for −717A>G and +1444C>T. In addition, to our knowledge, this is the first study addressing the relationship between all commonly studied *CRP* variants and CAD risk, facilitating a more precise evaluation and a better understanding of the role of *CRP* in CAD.

In conclusion, this study suggested that the *CRP* genetic variants previously reported to influence circulating C-reactive protein may not be linked to the development of CAD. This lack of association requires future larger studies to confirm or refute.
